# Sustained Viremia and High Viral Load in Respiratory Tract Secretions Are Predictors for Death in Immunocompetent Adults with Adenovirus Pneumonia

**DOI:** 10.1371/journal.pone.0160777

**Published:** 2016-08-17

**Authors:** Li Gu, Jiuxin Qu, Bing Sun, Xiaomin Yu, Hui Li, Bin Cao

**Affiliations:** 1 Department of Infectious Diseases and Clinical Microbiology, Beijing Chao-Yang Hospital, Beijing Institute of Respiratory Medicine, Capital Medical University, Beijing, China; 2 Department of Pulmonary and Critical Care. Beijing Chao-Yang Hospital, Beijing Institute of Respiratory Medicine, Capital Medical University, Beijing, China; 3 Department of Respiratory and Critical Care Medicine, Clinical Microbiology and Infectious Disease Lab, China- Japan Friendship Hospital, Beijing, China; Kliniken der Stadt Köln gGmbH, GERMANY

## Abstract

The predictors for fatal adenovirus (AdV) pneumonia among immunocompetent adults are unclear. Laboratory-confirmed, hospitalized AdV pneumonia adults were prospectively enrolled in Beijing Chao-Yang hospital from March to June 2013. Clinical data and serial whole blood and respiratory tract secretions from such patients were collected. Quantitative real-time polymerase chain reaction was performed to quantify the viral load. A total of 14 AdV pneumonia cases were consecutively enrolled, and four of them were fatal. Ten cases were caused by AdV-55, three by AdV-7 and one by AdV-3. There were no differences in age, gender or underlying diseases between the patients in the fatal cases and surviving cases. At admission (on day 5–7 after illness onset), the patients in fatal cases presented higher initial viral loads in respiratory tract secretions (8.578 ± 2.115 vs 6.263 ± 1.225 Log_10_ copies/ml, *p* = 0.023). All patients in fatal cases presented with viremia on day 12–14 (100% vs 66.7%, *p* = 0.017). A higher initial viral load in the respiratory tract and sustained viremia (more than 2 weeks) may be predictors for fatal clinical outcomes.

## Introduction

Severe adenovirus (AdV) infections causing significant acute respiratory distress syndrome have raised concerns for immunocompetent adults [1]. Most severe cases were previously reported to be associated with AdV-3, 4, 7 and 21 [2,3]. A new strain, AdV-55 (formerly known as AdV-11a), has been a major AdV pneumonia pathogen in immunocompetent adolescents and adults in China since 2008 [4]. Our previous study identified a fatal AdV-55 infection case with a patient who presented with systemic infection and high-level viremia [5]. However, even though AdV-55 as well as AdV-3, 4, 7 and 21 can cause severe cases, those strains are not necessarily associated with bad outcomes, and the pathogenesis of fatality is still unknown. We have already ascertained that AdV can be detected in whole blood specimens of severe cases, but we still lack the knowledge about the viral shedding history and the relationship between the viral clearance in the respiratory tract and viremia duration and clinical outcome. In this study, we focus on the dynamic virological changes in whole blood and respiratory tract secretions to see if lethal cases experienced a high viral load and longer duration of viral shedding compared to the non-lethal cases. We hypothesize that sustained virus shedding in the blood and/or respiratory tract can appear in severe immunocompetent adult cases, and it may be a risk factor for fatal outcome.

## Patients, Materials and Methods

The study was reviewed and approved by the institutional review board of Beijing Chao-Yang Hospital (the project approval number is 10-KE-49). Written informed consent was provided by all adults and the parents of patients aged less than 18 years. Adults with community-acquired pneumonia (CAP) (Age≥14yrs) admitted to Beijing Chao-Yang Hospital from March to June 2013 were prospectively included. Patients with HIV infection or neutropenia; receiving immunosuppressive chemotherapy or steroids equivalent to prednisone >15 mg/d for 30 days; who were pregnant or breast feeding women; or who were known or suspected to have active tuberculosis were excluded.

### Etiology Evaluation

Methods of etiological evaluation followed the standard for adults suspected with CAP [4–6]. In short, sputum or respiratory tract aspiration, blood and urine were collected at admission and submitted to the Infectious Disease and Clinical Microbiology Laboratory. Microbiological methods were based on the following tests: (1)Sputum specimens for Gram stain and cultures considered valid only if microscopy showed > 25 neutrophils and <10 epithelial cells per low field microscopy; (2)Urine specimens for the rapid detection of *S*. *pneumonia* and *Legionella pneumophila* antigen; (3) Blood culture; (4) Sputum and tracheal aspiration for virus real-time polymerase chain reaction (PCR) detection, including rhinovirus, influenza A and B, respiratory syncytial virus A and B, adenovirus, parainfluenza 1–4, coronavirus OC43 and 229E, and metapneumovirus; and (5) Sputum for *Mycoplasma pneumoniae* PCR detection. Only subjects with positive adenovirus results who were negative for other etiologies during the study period were enrolled.

### Clinical samples and data collection

The results of PCR testing for respiratory viruses were reported to clinicians within 6 hours after sputum collection. For those with positive AdV PCR testing, serial whole blood and respiratory tract samples were collected until death or discharge. In this study, we tried to collect the same type of respiratory tract samples from each patient to carry out serial viral load testing, to lower bias from different types of samples. For patients with mechanical ventilation, we collected serial tracheal aspirations, and for patients without mechanical ventilation, we collected sputum samples. We also collected data regarding age, gender, co-morbidities, clinical symptoms, vital signs, antimicrobial treatment, chest radiographic findings, and laboratory results. Recorded complications included the following: use of mechanical ventilation and extracorporeal membrane oxygenation (ECMO), and second bacterial or fungal infection. Patients were also followed up to discharge or death.

### Quantifying and Typing of Adenoviruses

Respiratory tract samples, including sputum and tracheal aspirations, were processed by equal volume 1% trypsin digestion. The viral DNA was extracted from 1 milliliter (ml) digested respiratory sample and 1 ml whole blood sample using a QIAamp DNA mini kit (Qiagen, Valencia, CA, USA). First, we amplified the entire hexon genes of the samples by PCR. The primer sequences are listed in Table 1 [7]. Then, the AdV type was determined using BLAST (http://blast.ncbi.nlm.nih.gov/Blast.cgi). Regarding the quantification of AdV in the samples, we used a commercial FQ-PCR kit (Daan Gene, Cat. #DA-B067, Guangzhou, China) targeting the DNA polymerase gene [GeneBank: KF279555.1]

**Table 1:** Primers used for amplification and sequencing of the entire hexon gene.

Primer	Sequence (5’-3’)	Position
Hexon-s	CCCGTCACCTTGGATTTGC	17942–17960
Hexon-as	CGATCATCCGAGAATCCAAA	21372–21391
H-1s	GCTTAACTTGCCTATCTGTG	18156–18175
H-1as	CCTATTGGGAGTCCTTCTTT	18774–18793
H-2s	AATGCTCCTGTAAAAGCT	18741–18758
H-2as	TGTAGTTGGGTCTGTTGG	19207–19224
H-3s	CAAGTTCCGAAGCTAAT	19171–19187
H-3as	ACCCTGTCCGATCTCAC	19591–19607
H-4s	AGATGAACTTCCCAACTACTGT	19466–19487
H-4as	ACTTGTATGTGGAAAGGCAC	19908–19927
H-5s	CGGACGTTATGTGCCTTTC	19898–19916
H-5as	GAGGGAGTTTCTTTGGTTT	20293–20311
H-6s	CCCATTTCCATTCCTTCTC	20232–20250
H-6as	AATTGACCTCATCAACCACC	20654–20673
H-7s	CAGGCAGGTGGTTGATG	20648–20664
H-7as	ATGGCACAGGCGAGCTTAT	21262–21280

### Statistical analysis

Two-tailed independent samples t-test or Mann-Whitney U-test (on condition of non-normal distributions) was used to compare continuous variables between the two groups. For the categorical data, univariate analysis was performed using the chi-square test or Fisher’s exact test. Significance was fixed at *p* value < 0.05. Data analysis was performed using SPSS 15.0 (SPSS Inc; Chicago, IL).

## Results

### Demographic characteristics

From March 2013 to June 2013, a total of 14 admitted cases were confirmed with adenovirus as the only pathogen of pneumonia; four of the patients died in the ICU. The mean age was 30 years old. Males predominated in number over females, with a sex ratio of approximately 6:1. Only two patients had co-morbidities, one with tuberculous pleuritis for 2 months and one with congenital heart disease. There were no differences in age, gender or underlying diseases between fatal cases and surviving cases. There are three types of AdV found in this study, AdV-55 (n = 10), AdV-7 (n = 3) and AdV-3 (n = 1). The AdV-55 ratio in the fatal group was similar to that in the surviving group (100% vs 60%, *p* = 0.251) (Table 2).

**Table 2:** Epidemiological and Clinical characteristics of patients with CAP caused by adenoviruses, comparison between survival and dead patients.

Characteristic	Survive (n = 10)	Death (n = 4)	*P* Value
Age, (Mean ± SD)	28.5±8.0	33.8±4.7	0.250
Male (%)	8/10 (80%)	4/4 (100%)	1.000
Underlying diseases (%)	1/10 (10%)	1/4 (25%)	0.505
Antibiotics before enrollment (%)	6/10 (60%)	4/4 (100%)	1.000
AdV-55 (%)	6/10 (60%)	4/4 (100%)	0.251
Days from onset of disease to initial viral PCR tests (Mean ± SD)	5.5±1.3	7.5±1.8	0.330
**Clinical features**			
PSI Score (Mean ± SD)	46.5± 26.7	101.3 ±8.0	0.002
Tmax (°C) (Mean ± SD)	39.7±0.6	39.5± 0.4	0.620
Dyspnea (%)	2/10 (20%)	4/4 (100%)	0.015
Hemoptysis (%)	1/10 (10%)	2/4 (50%)	0.176
Yellow sputum (%)	4/10 (40%)	0/4 (0%)	0.25
Diarrhea (%)	2/10 (20%)	1/4 (25%)	1.000
**Lab tests**			
White blood cells (Mean±SD) (10^9^/L)	4.8±1.7	4.9±3.6	0.934
Leukocyte<4000/mm^3^ (%)	4/10 (40%)	2/4 (50%)	1.000
Hemoglobin (Mean±SD) (g/L)	135.6±15.0	141.8±30.5	0.610
Platelet (Mean±SD) (10^9^/L)	132.3±51.8	56.0±31.4	0.019
AST (u/L) (Mean±SD)	124.2±111.6	189.0±54.5	0.297
ALT (u/L) (Mean±SD)	48.6±40.3	65.0±24.0	0.467
CK (Mean±SD)	1352.5±1429.0	1835.3±2442.0	0.647
LDH (u/L) (Mean±SD)	630.1±443.3	1572.8±234.8	0.002
PO_2_/FiO_2_ (Mean±SD)	355.3±46.6	151.1±97.0	0.020
Pleural effusion (%)	2/10 (20%)	3/4 (75%)	0.095
Both lungs involved (%)	4/10 (40%)	4/4 (100%)	0.085
Secondary infection (bacterial or fungal) (%)	2/10 (20%)	3/4 (75%)	0.09
ARDS (%)	1/10 (10%)	4/4 (100%)	0.032
Mechanical ventilation (%)	1/10 (10%)	4/4 (100%)	0.032
ECMO (%)	1/10 (10%)	3/4 (75%)	0.041
ICU admission (%)	1/10 (10%)	4/4 (100%)	0.005
LOS (Mean ± SD) (days)	13.3±9.2	13.5±6.5	0.969

Note: PSI = Pneumonia Severity Index. ARDS = Adult Respiratory Distress Symptom. LOS = Length of stay in hospital. ECMO = extracorporeal membrane oxygenation.

### Clinical Features: comparison between surviving and fatal cases

Most clinical symptoms and signs noted in the surviving cases and fatal cases had no differences, except for the pneumonia severity index (PSI) score and dyspnea. The fatal cases had a higher PSI score (101.3 ± 8.0) and dyspnea incidence (4/4) compared to the PSI score (46.5±26.7) and dyspnea incidence (2/10) in surviving cases (*p* = 0.002 and 0.015, respectively) (Table 2).

In laboratory findings, compared to surviving cases, the fatal cases had reports of lower platelet counts (56.0±31.4 vs 132.3±51.8, *p* = 0.019), higher lactate dehydrogenase (LDH) (1572.8±234.8 vs 630.1±443.3, *p* = 0.002) and decreased PaO_2_/FiO_2_ (151.1±97.0 vs 355.3±46.6, *p* = 0.02) (Table 2). All of the fatal cases described bilateral involvement on chest radiography, and 75% noted pleural effusion (Table 2). For the surviving cases, 40% (4/10) noted bilateral involvement, and 20% (2/10) described pleural effusion. There were no significant differences between the two groups in bilateral involvement or pleural effusion.

### The AdV load tracking in the respiratory tract samples and whole blood

**Fig 1:**
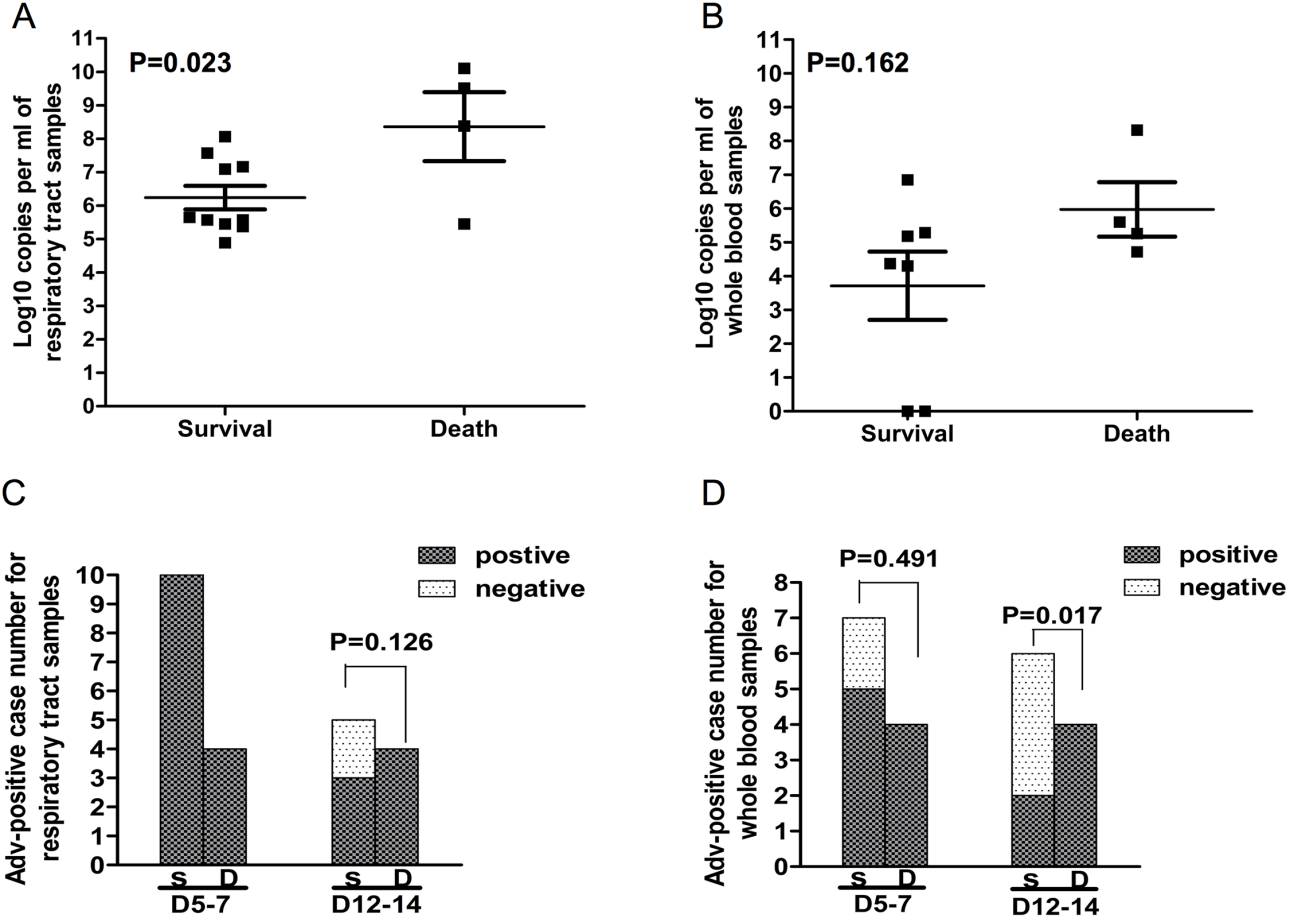
The AdV tracking in the respiratory tract samples and whole blood. **(A)** AdV viral load of respiratory tract samples on day 5–7 days after disease onset between survival cases and fatal cases. **(B)** AdV viral load of whole blood on day 5–7 days after disease onset between survival cases and fatal cases. Viral load level is presented with log_10_ DNA copy numbers per ml of sample on the Y-axis. **(C)** The comparison of Adv DNA positive ratio for respiratory tract samples between survival cases and fatal cases on day 5–7 days after disease onset and on day 12–14 after disease onset. **(D**) The comparison of Adv DNA positive ratio for whole blood between survival cases and fatal cases on day 5–7 days after disease onset and on day 12–14 after disease onset. The y-axis represents case numbers of positive and negative viral shedding. S = survival cases. D = fatal cases. D5-7 = on day 5–7 days after disease onset. D12-14 = on day 12–14 after disease onset.

The mean time from disease onset to initial PCR test for respiratory tract samples and whole blood was 6.23 days, and there were no significant difference between the fatal group (7.5±1.8days) and surviving group (5.5±1.3days). We measured the adenoviral load in respiratory tract samples and whole blood, expressed as log_10_ DNA copies per ml samples. For initial viral load in respiratory tract samples on day 5–7 after disease onset, the results showed that, compared to the surviving cases, the fatal cases had a higher initial viral load (8.578 ± 2.115 vs 6.263 ± 1.225, *p* = 0.023) (Fig 1A). However, for the initial viral load in whole blood, there was no significant difference between the two groups (3.71±2.67 vs 5.978±1.610, *p* = 0.162, Fig 1B).

We also investigated viral shedding duration evaluated by positive ratio on day 5–7 and on day 12–14 after illness onset. For respiratory tract samples, there was no significant difference of viral positive ratio between the two groups either on day 5–7 (100% vs 100%) or on day 12–14 after onset of disease (60% vs 100%, *p* = 0.126) (Fig 1C). Viremia was common at admission (on 5–7 day after onset of illness), as noted in 100% (4/4) of the fatal cases and 71.4% (5/7) of the surviving cases (*p* = 0.491). On day 12–14 after onset of illness, however, it persisted in 100% (4/4) of the fatal cases with 33.3% (2/6) of the surviving cases (*p* = 0.017) (Fig 1D).

**Fig 2:**
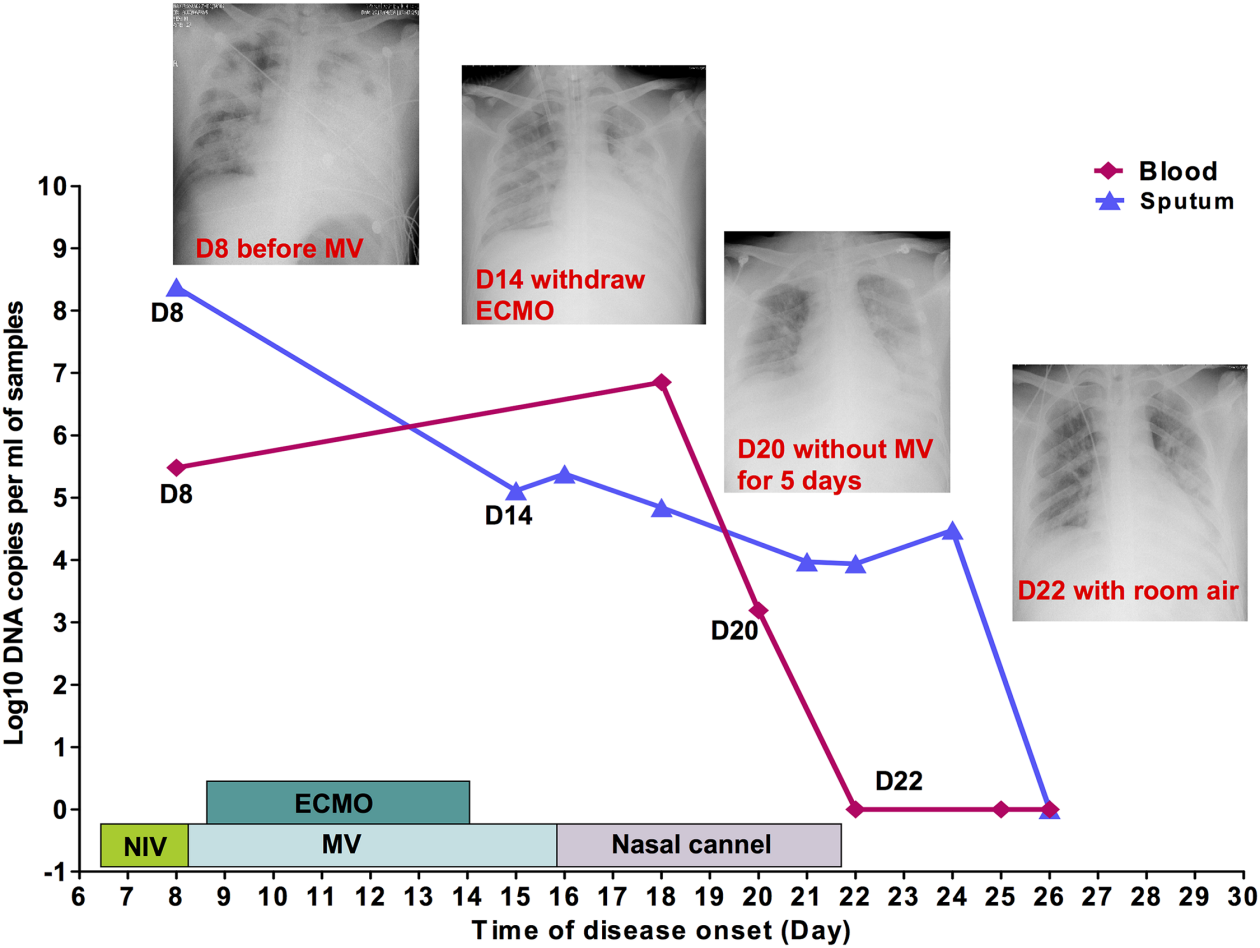
The kinetics of Adv loads in serial respiratory tract samples and whole blood and parallel respiratory support treatment, chest X-ray and clinical outcomes. Viral load level is presented with log_10_ DNA copy numbers per ml of sample on the y-axis. A 25-year-old male survival patient with ARDS. NIV = noninvasive ventilation, High F V = high-frequency ventilation.

For the surviving severe patients, we found that the clinical manifestation recovered gradually with a downward trend in viral load in respiratory tract and whole blood samples. As shown in Fig 2, a 25-year-old male with acute respiratory distress syndrome (ARDS) had a high initial viral load (10^8.32^ copies/ml) in tracheal aspiration. His condition improved, with the sputum viral load going down on day 14, and ECMO was withdrawn on day 14. With the viral load in blood going down to negative on day 22, the clinical condition was significantly improved, and the patient did not need oxygen therapy; the chest x-ray was also remarkably resolved.

**Fig 3:**
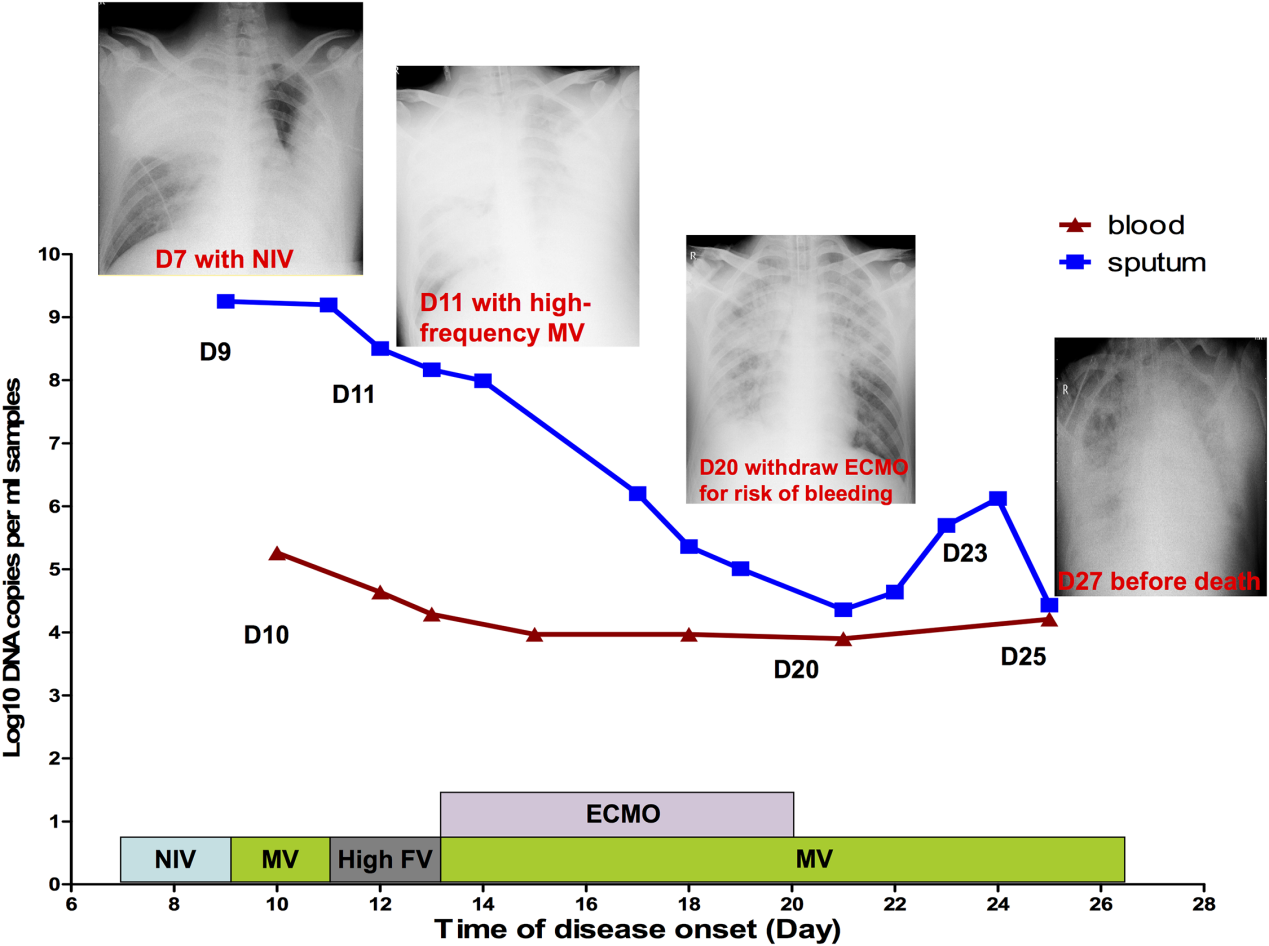
The kinetics of Adv loads in serial respiratory tract samples and whole blood and parallel respiratory support treatment, chest X-ray and clinical outcomes. Viral load level is presented with log_10_ DNA copy numbers per ml of sample on the y-axis. A 34-year-old male fatal patient with ARDS. NIV = noninvasive ventilation, High F V = high-frequency ventilation.

As shown in Fig 3, the respiratory tract viral load of a 34-year-old male with ARDS also had a high initial viral load (10^9.25^ copies/ml) in tracheal aspiration. The viral load in tracheal aspiration gradually decreased during the first 20 days, and the lung infiltrate absorbed partially. However, the viral load trended up again and the patient died. He also maintained a viral load in whole blood before death.

### Complications, Management and Prognosis of patients

**Fig 4:**
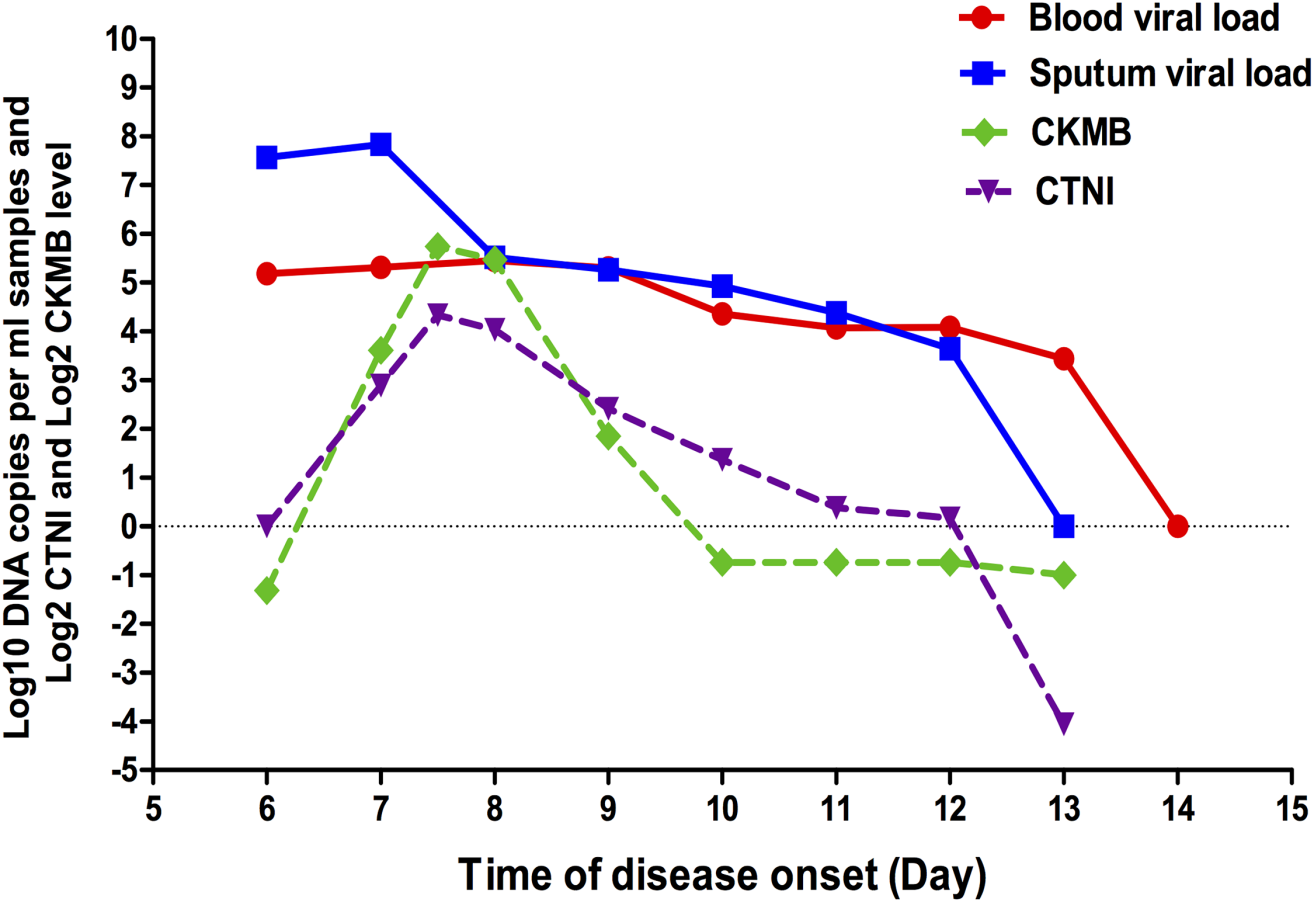
The kinetics of Adv loads in sequential sputum and whole blood, CK-MB and CTNI level from a 29-year-old male survival patient with myocarditis. On the y-axis, viral load level is presented with log_10_ DNA copy numbers per ml of sample, and myocardial enzyme level is expressed as log_2_ CK-MB ng/ml and log_2_ CTNI ng/ml. CK-MB = Creatine Kinase-MB, CTNI = Cardiac troponin I.

Antibiotics were given to all of the patients empirically before and after confirmed diagnosis. There is currently no formally approved antiviral therapy for the treatment of severe life-threatening adenovirus infection in China. Acyclovir, ganciclovir or ribavirin is commonly chosen by the physician to treat adenoviral infection. In this study, all of the fatal patients were administered antiviral drugs—one was treated with ganciclovir, two with acyclovir and one with ribavirin. In surviving patients, 50% were treated with antiviral drugs—three with ganciclovir, one with acyclovir and one with ribavirin. All of the fatal patients (4/4) were complicated with ARDS and admitted to the ICU. They needed mechanical ventilation, and three of them received ECMO to maintain oxygenation. For surviving patients, one of them (1/10) was admitted to the ICU due to ARDS, and had mechanical ventilation and ECMO; one patient with respiratory failure was treated with non-invasive ventilation, and one patient was treated for myocarditis. The myocarditis patient presented with peak levels of creatine kinase isoenzyme (CK-MB) and cardiac troponin I (CTNI) on day 7–8 after disease onset, and eight days later, with viral load going down to negative, CK-MB and CTNI went down to normal levels in parallel (Fig 4).

For the four fatal patients, the times of death were on days 14, 16, 22 and 28 after disease onset, respectively. There was no significant difference in length of stay in-hospital between the two groups (13.5±6.5 days vs 13.3±9.2 days, *p* = 0.969) (Table 2).

### Superinfection

**Fig 5:**
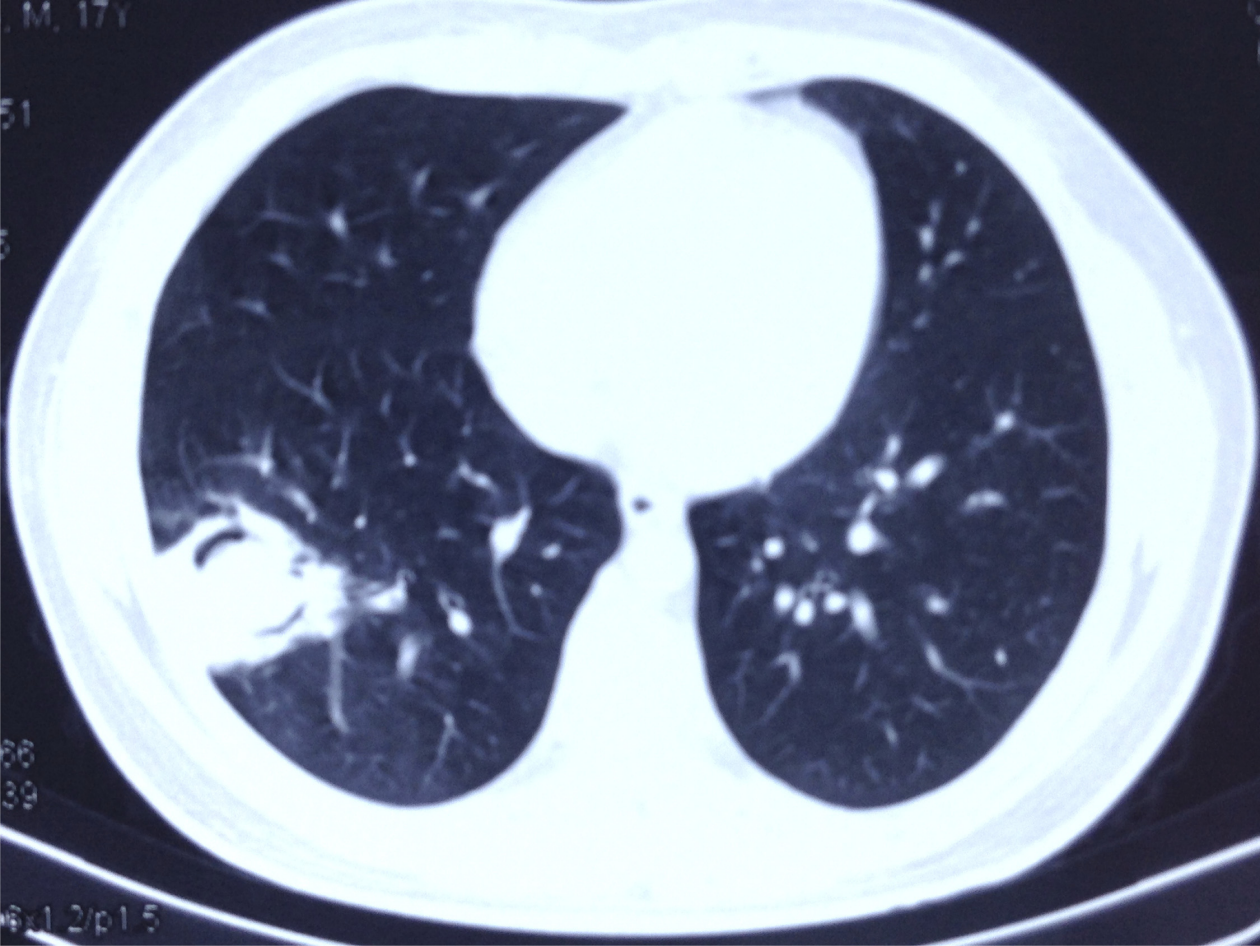
Chest CT of a 16-year-old male survival patient. On day 40 after disease onset, chest CT showed a typical crescent sign and halo sign indicative of invasive pulmonary aspergillosis. The patient recovered from invasive pulmonary aspergillosis after voriconazole treatment for 12 months.

There were three patients complicated with bacterial or fungal infections among the fatal cases. The first patient presented with consecutive *aspergillus fumigatus* in tracheal aspirate cultures, the second patient had a cavity present on chest CT, and the third patient had *Acinetobacter baumannii* in tracheal aspirate and pleural effusion cultures. There were only two patients complicated with superinfections in surviving patients. One patient (a 16-year-old male) presented with a typical crescent sign and halo sign with chest CT follow-up; voriconazole was administered to resolve this condition (Fig 5). Another patient with ECMO presented with *Acinetobacter baumannii in* tracheal aspirates.

## Discussion

We investigated the relationship between the virological factors and clinical outcomes in a cohort of 14 hospitalized adults with AdV pneumonia. Our results suggest that a higher initial viral load (10^8^ copy/ml) in the respiratory tract samples on day 5–7 after disease onset is a predictor for fatal clinical outcome. We also reported that viremia is common and sustained viremia for 14 days or more may be associated with mortality

The pathogenesis of mortality in Adv pneumonia is still unknown. Virological factors, e.g., a new strain with new genetics, viral load, slow virus clearance and systemic infection with viremia likely play key roles for severe Adv [2–8]. However, no study has evaluated the viral shedding history among immunocompetent adults with AdV pneumonia. This study first monitored the consecutive viral load in respiratory tract samples and whole blood samples. Our previous clinical study demonstrated that on day 5–7 after disease onset, the peak stage of illness presented for patients with shortness of breath or severe dyspnea[5]. Again, in this study, we showed that the viral load on day 5–7 could also provide an insight into the severity of illness. Evidence even proved that a higher level of viral load in respiratory tract samples on day 5–7 after disease onset was significantly associated with fatal outcome.

We have noted that viremia is quite common on day 5–7 after disease onset, when 9 out of 11 (81.8%) patients had viremia. Adenovirus viremia has been found in hematopoietic stem cell transplantation recipients and associated with AdV disease [8]. Compared with previous reports of viremia and clinical outcomes, another novel finding is that we demonstrated that fatal outcomes could be predicted by sustained viremia, but not by viremia itself. In this study, we showed that 100% (4/4) of patients in fatal cases presented with viremia on day 12–14 after disease onset, compared with 60% (*p* = 0.126) of the patients in surviving cases.

In one case, as shown in Fig 2, even though the patient presented with a higher viral load (10^8.32^ copies / ml) in tracheal aspiration, which may be associated fatal outcome, his clinical manifestation recovered gradually with a downward trend in the viral load in respiratory tract and whole blood samples. Compared to this case in Fig 3, the patient described in Fig 3 not only had a higher viral load (10^9.25^ copies/ml) in tracheal aspiration but also presented with sustained elevated viral copies, especially in whole blood. Shike et al. also reported a 6-month-old infant with systemic infection by adenovirus who had high-level viremia and showed reduction in viral load paralleling her clinical recovery[9]. Therefore, in severe cases, dynamic monitoring of viral shedding, especially in whole blood, could help predict the clinical outcome. Patients might have bad outcomes if the viral load in whole blood does not present a significant downward trend around two weeks after disease onset.

There is currently no formally approved antiviral therapy for the treatment of severe life-threatening adenovirus infection in China. Cidofovir is considered the medicine of choice for severe infection in immunocompromised patients. Cidofovir is not available in most hospitals in China, including our hospital. Acyclovir, ganciclovir or ribavirin is usually prescribed in China. In this study, antiviral drugs were administered in all of the fatal cases—one patient was treated with ganciclovir, two with acyclovir and one with ribavirin. In surviving patients, 50% were treated by antiviral drugs—three with ganciclovir, one with acyclovir and one with ribavirin. The choice of the antiviral medicine was decided by the patient’s physician. As none of these three medicines have been confirmed to be effective for AdV infection, the relationship between viral shedding and clinical outcomes in this study was not associated with anti-adenoviral treatment effect.

Our study has two limitations. As AdV 55 was the most common infection type (10/14, 71.4%) in this study, results might be more significant in AdV 55-associated pneumonia and might not be generalizable to other types of AdV pneumonia. In our previous study, adults infected with AdV 55 were 10 years older and presented with higher PSI scores compared with adults infected with other serotypes [4]. Another limitation of this descriptive work may be the small number of analyzed patients, especially in the group of fatal cases (n = 4). More cases are needed to confirm our findings.

In conclusion, our data provide new insight into the virology of AdV pneumonia. A higher initial viral load (10^8^ copy/ml) in the respiratory tract on day 5–7 after disease onset and sustained viremia for 2 weeks or more may be associated with fatal clinical outcomes.
